# Sexual Transmission of Arboviruses: A Systematic Review

**DOI:** 10.3390/v12090933

**Published:** 2020-08-25

**Authors:** Bradley J. Blitvich, Tereza Magalhaes, S. Viridiana Laredo-Tiscareño, Brian D. Foy

**Affiliations:** 1 Department of Veterinary Microbiology and Preventive Medicine, College of Veterinary Medicine, Iowa State University, Ames, IA 50011, USA; viri@iastate.edu; 2 Arthropod-Borne and Infectious Diseases Laboratory, Department of Microbiology, Immunology and Pathology, Colorado State University, Fort Collins, CO 80523, USA; Tereza.Magalhaes@colostate.edu (T.M.); Brian.Foy@ColoState.edu (B.D.F.)

**Keywords:** arbovirus, sexual transmission, *Asfarviridae*, *Bunyavirales*, *Flaviviridae*, *Orthomyxoviridae*, *Reoviridae*, *Rhabdoviridae*, *Togaviridae*

## Abstract

Arthropod-borne viruses (arboviruses) are primarily maintained in nature in transmission cycles between hematophagous arthropods and vertebrate hosts, but an increasing number of arboviruses have been isolated from or indirectly detected in the urogenital tract and sexual secretions of their vertebrate hosts, indicating that further investigation on the possibility of sexual transmission of these viruses is warranted. The most widely recognized sexually-transmitted arbovirus is Zika virus but other arboviruses, including Crimean-Congo hemorrhagic fever virus and dengue virus, might also be transmitted, albeit occasionally, by this route. This review summarizes our current understanding on the ability of arboviruses to be sexually transmitted. We discuss the sexual transmission of arboviruses between humans and between vertebrate animals, but not arthropod vectors. Every taxonomic group known to contain arboviruses (*Asfarviridae*, *Bunyavirales*, *Flaviviridae*, *Orthomyxoviridae*, *Reoviridae*, *Rhabdoviridae* and *Togaviridae*) is covered.

## 1. Introduction

Viruses that cycle between blood-feeding arthropods and susceptible vertebrate hosts are known as arthropod-borne viruses (arboviruses) [1]. Arboviruses are usually transmitted by mosquitoes and ticks but other arthropod vectors include midges and sandflies. Most recognized arboviruses are classified within three taxonomic groups: the order *Bunyavirales* and the families *Flaviviridae* and *Togaviridae*. All other known arboviruses belong to the families *Asfarviridae*, *Orthomyxoviridae*, *Reoviridae* and *Rhabdoviridae*.

Although arboviruses primarily cycle between arthropod vectors and vertebrate hosts, other modes of transmission have been recognized [1,2,3]. Some arboviruses have been directly transmitted between humans by organ transplantation, blood transfusion, breast-feeding and intrauterine transmission. Humans can also become infected through the inhalation of aerosols, needle-stick injury and direct contact with infected animals or animal products. Non-human vertebrates can also acquire arbovirus infections through non-vector-borne transmission. The aforementioned modes of non-arthropod-borne transmission are usually rare, having minimal impact on arbovirus maintenance in nature [2,3].

Another mode of non-vector-borne arbovirus transmission, one that has gained considered interest in recent years, is sexual transmission. This recent interest is largely due to the discovery that Zika virus (ZIKV), a mosquito-transmitted virus belonging to the family *Flaviviridae*, can be sexually transmitted between humans, with dozens of sexually acquired infections now documented [4,5,6]. Many review articles have been written on the subject. Most of these articles have focused exclusively on ZIKV, but a few encompass the entire family *Flaviviridae* and, occasionally, arboviruses from the order *Bunyavirales* and family *Togaviridae* [5,7,8,9,10,11,12,13,14]. Here, we provide a review on the urogenital localization of arboviruses and their potential to be sexually transmitted between vertebrates and include, for the first time, every taxonomic group that contains arboviruses (Table 1 and Table 2). We summarize information from case reports, disease outbreaks, surveillance studies and experimental infection studies and describe which arboviruses have been detected in the male and female reproductive tracts and documented to be shed in sexual bodily fluids such as semen and vaginal secretions.

### 1.1. Asfarviridae

African swine fever virus (ASFV) is the sole member of the genus *Asfivirus* (family *Asfarviridae*) and only known DNA arbovirus [15]. The virus is maintained in nature in a sylvatic transmission cycle between soft ticks and warthogs and is a cause of disease in domestic pigs. A review article by Thacker and colleagues explains that ASFV has been isolated from the semen of an experimentally infected boar and was transmitted by artificial insemination to a recipient sow [16]. However, a personal communication was cited, and there is no peer-reviewed data to support this finding.

### 1.2. Bunyavirales

In 2017, the International Committee on Taxonomy of Viruses reclassified the family *Bunyaviridae*, formerly divided into five genera, elevating it to the order *Bunyavirales*. This recently established order contains 12 families, three of which contain recognized arboviruses, and these are the *Nairoviridae*, *Peribunyaviridae* and *Phenuiviridae* [17].

### 1.3. Nairoviridae

The family *Nairoviridae* includes Crimean-Congo hemorrhagic fever virus (CCHFV; genus *Orthonairovirus*), a tick-borne pathogen that can cause fatal hemorrhagic fever in humans [18]. Human infections also occur by contact with blood or tissues from viremic livestock, with slaughterhouse workers and butchers considered high-risk occupational groups. Several cases of Crimean-Congo hemorrhagic fever (CCHF) are suspected to have occurred after sexual contact between spouses [19,20,21]. In 1999, a woman in Iran developed CCHF with sexual transmission considered the most likely route of infection [21]. Her husband (a physician) had recently recovered from CCHF, acquiring the virus after treating the index patient. Another case occurred in Turkey in 2008 when a woman developed CCHF after having sexual intercourse with her husband, a convalescent CCHF patient recently discharged from the hospital [19]. Three more CCHF cases suspected to have been sexually acquired occurred in Russia in 2005, 2010 and 2014 [20]. One secondary case (a woman) had sexual contact with her infected husband four to seven days after he developed a relatively mild case of CCHF. The other secondary cases had sexual contact with their infected spouses one to two days before the primary cases developed symptoms. Sexual transmission was considered the most likely route of infection for all of the aforementioned secondary cases, even though they all occurred in CCHF endemic regions. Some secondary cases had no recent history of tick bite or direct contact with animals, suggesting that these routes of infection were unlikely. However, close contact between human skin and mucosal surfaces could not be excluded as a route of infection.

Subsequent studies have provided evidence that CCHFV disseminates to the human genital tract. A case report describes the occurrence of epididymo-orchitis in a CCHF patient in Turkey [22]. The patient experienced sudden pain and swelling in his right testis four days after illness onset. The left testis was unaffected. CCHFV RNA persisted in urethral and vaginal swabs from CCHF patients for 9 and 11 days, respectively, after illness onset [23]. Viral RNA copy numbers were often higher in genital swabs than sera collected on the same day. Experimental infection studies revealed that CCHFV persists in the testes of infected cynomolgus macaques [24]. Unilateral inflammation occurred in the testis of some animals, and viral RNA and antigen were present in the seminiferous tubules, the site of sperm production. Nairobi sheep disease virus (genus *Orthonairovirus*) replicates in lamb testis cell cultures, but evidence that it disseminates to the testes of sheep is lacking [25].

### 1.4. Phenuiviruses

The family *Phenuiviridae* includes Heartland virus (HRTV), severe fever with thrombocytopenia syndrome virus (SFTSV) and Rift Valley fever virus (RVFV). HRTV (genus *Banyangvirus*) is a tick-borne virus associated with sporadic cases of severe and sometimes fatal human disease in the United States [26,27]. A post-mortem examination of a HRTV patient who died in 2015 revealed the presence of viral antigen in multiple tissues, including the testes [28]. Congestion and focal hemorrhage of the testis were observed. SFTSV (genus *Banyangvirus*) is a tick-borne virus that causes life-threatening human disease in Asia [29]. SFTSV RNA was detected once in the semen of a patient in Japan in 2018 collected 30 days after illness onset [30]. RVFV (genus *Phlebovirus*) is a mosquito-transmitted pathogen of ruminants and humans in sub-Saharan Africa and the Arabian Peninsula [31]. RVFV RNA persisted for at least 117 days in the semen of an immunocompromised patient from France who had recently visited Mali [32]. Virus isolation was unsuccessful. It is not clear from these case reports whether the detection of *Phenuivirus* RNA/antigens in the patients’ semen or testes was due to the severity of disease or suggestive of the normal potential for sexual transmission of these viruses.

### 1.5. Peribunyaviruses

The family *Peribunyaviridae* contains four genera, the largest being the genus *Orthobunyavirus* which includes Schmallenberg virus (SBV), Akabane virus (AKAV) and Aino virus (AINOV), three *Culicoides*-transmitted viruses that cause pregnancy loss and congenital defects in ruminant livestock [33,34]. Infectious SBV has been isolated from the semen of naturally infected bulls [35,36]. Other studies have reported the detection of SBV RNA in the semen of naturally and experimentally infected bulls, sometimes for as long as three months [35,37,38]. SBV RNA was not detected in the semen of experimentally infected goats, although only two were tested, but was detected in ovarian tissue of a goat and sheep [39,40].

AKAV could not be isolated from the semen of experimentally infected bulls [41]. The virus also could not be isolated from vaginal swabs taken from cows that were artificially inseminated at estrus with uninfected semen then challenged with virus by intra-uterine inoculation, although isolates were recovered from uterine and ovarian tissues [42]. In another study, ejaculates were collected from several bulls naturally infected with AKAV, as demonstrated by the presence of virus or virus-specific antibodies in their blood [43]. Ejaculates were inoculated into sheep and two of 16 seroconverted. One of six sheep inoculated with ejaculates from bulls naturally infected with AINOV also seroconverted. However, the authors could not dismiss the possibility that the sheep acquired natural AKAV and AINOV infections by insect bite during the course of the study.

### 1.6. Flaviviridae

The family *Flaviviridae* contains four genera, including the genus *Flavivirus*. Most viruses belonging to this genus are recognized arboviruses, and examples include dengue virus (DENV), Japanese encephalitis virus (JEV), West Nile virus (WNV), yellow fever virus (YFV) and ZIKV, all of which are mosquito-transmitted viruses of global public health significance [44,45]. Flaviviruses of localized public health concern include tick-borne encephalitis virus (TBEV), a tick-borne pathogen that occurs primarily in Asia, Europe and Russia [46].

### 1.7. Zika Virus: Sexual Transmission between Humans

It is well documented that ZIKV can be sexually transmitted between humans [7,8,9,10,11,12,13,14]. The first evidence of sexual transmission was provided in a case report that describes a woman in the United States in 2008 with no recent travel history who developed a symptomatic ZIKV infection after sexual intercourse with her husband, a convalescent ZIKV patient who had recently returned from Senegal [4]. Both diagnoses were based on serologic data, and virus was not recovered from either patient. Among the symptoms reported in the male patient was hematospermia. Many cases of sexually acquired ZIKV infection have since been reported [5,7,8,9,10,11,14]. The first study to sequence the complete ZIKV genomes from the index and secondary patients describes a woman in France who developed ZIKV symptoms in 2016, shortly after several sexual encounters with the index patient, a male who had recently traveled to Brazil [47]. The woman had never visited a region with active ZIKV transmission. Sexual transmission of ZIKV has now been documented in at least 13 countries without concurrent vector-borne transmission [10].

The majority of sexually acquired ZIKV infections have been transmitted from male to female partners, although female-to-male and male-to-male transmission has been reported [7,56,57,58]. Most infections have occurred from unprotected vaginal sex, but transmission through anal sex and potentially oral sex has been described [7,47,57]. Both symptomatic and asymptomatic individuals can transmit ZIKV through sexual contact. Some studies have shown that in situations where both sexual partners become symptomatic, secondary patients usually develop symptoms within 12 days from the occurrence of symptoms in the index patient, although up to 44 days has been reported [7,59]. Some modeling studies have provided evidence that sexual transmission alone cannot maintain ZIKV in a human population [73,74,75,76]. In one study, the basic reproduction number (R0) was calculated as 2.1, with the contribution of sexual transmission estimated to be 3.0% [75]. However, other modeling studies have predicted a much higher contribution, sometimes by combining vector transmission with sexual transmission or by considering heterogeneity in sexual transmission among the human population, with the contribution of sexual transmission ranging from 14% to 46% [77,78]. Accordingly, recent household-based studies in Puerto Rico and Brazil showed that sexual partners within households are at a higher risk of being exposed to ZIKV, suggesting that sexual transmission is an important contributor of ZIKV transmission dynamics in endemic regions [79] (Magalhaes et al., unpublished).

The first isolation of ZIKV from semen was from a patient in Tahiti in 2013 [60]. The virus has since been isolated from the semen of many other patients, persisting up to 69 days after illness onset [61]. Multiple studies have reported prolonged shedding of ZIKV RNA in the semen of patients, and this has been documented for both symptomatic and asymptomatic infections [62,63,64,80,81,82,83,84]. For example, ZIKV RNA was detected in the semen of 48 of 112 (43%) patients at 31–60 days after illness onset, persisting for 281 days in one patient [63]. Seventy-eight ZIKV RNA-positive semen samples from 46 patients were tested by virus isolation in cell culture, with virus recovered from 3 of 19 (15.8%) samples collected within 30 days of illness onset, but none from the 59 samples collected at later times. In another study, ZIKV RNA was detected in the semen of an immunocompromised patient at 941 days after illness onset [62]. Additionally, ZIKV RNA has been detected in the anorectal mucosa of naturally infected male and female patients [85]. Rectal swabs were collected from 10 patients and all were positive for ZIKV RNA. One swab also contained infectious virus. ZIKV RNA has also been detected in vaginal and cervical secretions, persisting for as long as 180 and 31 days, respectively [65,66,67,68,69,70,71]. The ability of ZIKV to occur at a higher frequency and to persist for longer in the testis and semen compared to vaginal and cervical secretions could explain why male-to-female transmission is more common that female-to-male transmission [86]. The World Health Organization recommends that men and women returning from areas with active ZIKV transmission practice abstinence or avoid unprotected sex for at least three and two months, respectively [87].

Human germ cells are highly susceptible to ZIKV infection and replication, as determined in experiments where human testicular biopsy samples were infected ex vivo, with virus continuously produced for at least 59 days with no substantial decrease in titer [88,89]. ZIKV infection had no apparent effect on the morphology, cell viability or hormonal production of the tissue explants [88]. Treatment with berberine chloride, a plant-derived alkaloid, significantly reduced the ex vivo replication of ZIKV, suggesting that this compound could be used to prevent ZIKV sexual transmission [89].

### 1.8. Zika Virus: Mouse Models to Study Sexual Transmission

Mouse models have been developed to study the sexual transmission, tissue tropisms and shedding kinetics of ZIKV [90,91,92,93]. These experiments have been performed using mice with genetically deficient interferon responses or antibody/drug-induced immunocompromisation because ZIKV cannot replicate and cause disease in immunocompetent mice. Some studies have demonstrated that at least 50% of naïve females become infected after naturally mating with infected males [90,93]. Coitus free sexual transmission has also been reported in experiments where female mice were inoculated with virus by the intravaginal route [94,95]. Several studies have reported the isolation of ZIKV from at least 60% of ejaculates from infected mice, with the virus persisting for at least 23 days after inoculation [93,96,97]. ZIKV infects and damages the testes of infected mice and can cause infertility [92,93,98,99,100,101,102,103,104]. ZIKV also replicates in the female reproductive tract depending on the immunocompromised status of the females and timing of infection relative to the estrus cycle, having been detected in uterine tissue, ovaries and vaginal washes [95,105,106,107]. Mice challenged by the anorectal route are also susceptible to infection, with virus detected in multiple organs, including the testes, indicating that anal intercourse is a possible mode of ZIKV transmission [108,109].

### 1.9. Zika Virus: Non-Human Primate Models to Study Sexual Transmission

Several non-human primate (NHP) models have been developed to study ZIKV replication, transmission and pathogenesis [110,111,112,113]. Viremia and seroconversions were detected in rhesus and cynomolgus macaques after intravaginal and intrarectal inoculation, providing evidence that ZIKV is sexually transmitted [114]. Several groups have reported the shedding of ZIKV RNA in sexual fluids of infected NHPs [115,116,117]. In one study, ZIKV RNA was detected at high levels in the semen of male NHPs, remaining detectable for 28 days, with viral RNA also present but at lower levels in vaginal secretions of female NHPs [115]. ZIKV has been detected throughout the reproductive tracts of infected male and female NHPs [115,116,118,119,120,121,122,123]. ZIKV RNA was detected in the testes and epididymis of male olive baboons for up to 10 and 41 days after infection [116]. ZIKV RNA was also detected in multiple tissues of the reproductive tract (cervix, ovaries, uterus and vagina) of female rhesus macaques [121]. Additionally, ZIKV RNA has been detected throughout the maternal and fetal/neonatal reproductive tracts of rhesus macaques after pregnant females were subcutaneously inoculated with virus [118].

### 1.10. Zika Virus: Other Animal Models to Study Sexual Transmission

Evidence of ZIKV infection has been detected in the reproductive tracts of several non-murine, non-primate vertebrate species [124,125,126,127]. ZIKV RNA was detected in the testes of tree shrews (*Tupaia belangeri*) after subcutaneous inoculation [127]. Another study reported the presence of ZIKV antigen in the testes of intradermally inoculated Jamaican fruit bats (*Artibeus jamaicensis*) [126]. ZIKV antigen was also detected in the testes of immunocompetent guinea pigs after subcutaneous and intranasal inoculation [124]. A more recent study demonstrated that immunocompetent guinea pigs are susceptible to ZIKV infection after vaginal inoculation, with viral RNA detected in various tissues including the ovaries and uterus [125]. Infectious virus persisted in vaginal secretions for at least 21 days.

### 1.11. Dengue Virus

Probable sexual transmission of DENV occurred in South Korea in 2013 [48]. The index patient was a woman who had recently visited Indonesia and developed dengue on the day of her return. The secondary patient was a man who had not traveled internationally for over a year. He had sexual intercourse with the woman the day she arrived home and developed dengue nine days later. DENV is not endemic in South Korea, and sexual transmission was considered the most likely route of infection for the man. Another case of dengue assumed to be acquired by sexual transmission occurred in Spain in 2019 [49]. Both patients were male, and both contained DENV sequence in their semen. DENV is not endemic in Spain, and only one of the two patients had recently traveled, visiting Cuba and the Dominican Republic, two countries where DENV is endemic.

DENV RNA was detected by quantitative RT-PCR in vaginal secretions from a female patient and the semen of a male patient for up to 18 and 37 days, respectively, after illness onset [50,51]. The semen also contained negative-sense DENV RNA, providing evidence that the genital tract is a site of active virus replication [51]. A recent article questioned whether the detection of DENV RNA in the semen of the male patient was due to RT-PCR cross-contamination because some features of the case were atypical, most notably the detection of DENV RNA in the patient’s serum 9 days after illness onset because it usually clears after 4-6 days of illness [128]. The vaginal secretions reported to contain DENV RNA were tested in the same laboratory as that which tested the semen. In another study, DENV RNA was not detected in the semen of five men with acute infections [129].

### 1.12. West Nile Virus

In 2014, a woman in the United States developed neuroinvasive WNV disease, despite recalling no recent mosquito exposure [52]. The authors considered sexual transmission the most likely route of infection even though the patient lived in a WNV endemic area. In another study, WNV RNA was detected by quantitative RT-PCR in the semen of a male patient, but the cycle threshold value was close to the diagnostic criterion [53]. WNV antigen has been detected postmortem in the testis of an immunocompromised patient [54]. WNV antigen has also been detected in multiple tissues, including the ovaries and testes, of deceased parrots [130]. Mice inoculated by the vaginal route developed fatal WNV infections [131].

### 1.13. Other Flaviviruses

There is evidence to suggest that other flaviviruses can infect the vertebrate reproductive tract and persist in sexual fluids. YFV RNA was detected in the semen of a patient at 21 days after illness onset [55]. JEV persisted in the semen of an experimentally infected boar for 17 days and female recipients became infected after artificial insemination [132]. JEV also infects and cause inflammatory changes in the testes of JEV-infected boars [133]. Spondweni virus (SPONV), the closest known relative to ZIKV, was detected in the reproductive tract (testes, epididymides and seminal vesicles) of interferon-deficient mice at titers similar to that of ZIKV [96]. Two of 50 (4%) ejaculates from SPONV-infected mice contained virus, which is significantly lower than the 60–72% reported for ZIKV, indicating that SPONV might be sexually transmitted between immunocompromised mice, but at a much lower efficiency than ZIKV. Naïve female mice became infected with TBEV after mating with infected males, with viral RNA detected in embryonal tissues of two of 11 litters [134]. Tembusu virus, a mosquito-transmitted pathogen of poultry, replicates in the ovaries and testes of infected birds [135,136,137,138].

### 1.14. Orthomyxoviridae

There are seven genera in the family *Orthomyxoviridae*, including two that contain arboviruses. Viruses in the genus *Quaranjavirus* appear to cycle primarily between soft ticks and aquatic birds [139]. Examples include Quaranfil and Wellfleet Bay viruses [139]. Viruses in the genus *Thogotovirus* cycle between hard ticks and mammals [140]. Examples include Bourbon and Thogoto viruses. There is no experimental evidence to suggest that any arboviruses in these genera could be sexually transmitted between vertebrates. In one study, Wellfleet Bay virus was not recovered from the gonads of experimentally infected birds [141].

### 1.15. Reoviridae

The family *Reoviridae* contains 15 genera. Viruses in three genera (*Coltivirus*, *Orbivirus* and *Seadornavirus*) replicate in both arthropod vectors and vertebrate hosts. The type species of the genus *Orbivirus* is bluetongue virus (BTV), a *Culicoides*-transmitted virus that affects ruminants [142]. There have been many reports describing the isolation of BTV from the semen of experimentally infected bulls [143,144,145,146,147]. In one study, BTV was recovered from the semen of 7 of 20 bulls after virus challenge, with isolations made only from ejaculates collected during the period of viremia [144]. Isolations of BTV from the semen of naturally infected bulls have also been reported but are not common [148,149]. Cows artificially inseminated with BTV-contaminated semen develop viremias and seroconvert [144,150]. However, cows did not become infected after naturally mating with an infected bull [151]. In studies performed on sheep, BTV RNA was detected in the semen of naturally infected rams and remained detectable for up to 116 days after symptom onset [152]. BTV has been isolated from the testes of experimentally infected rams, and testicular degeneration was observed in rams after experimental and natural infection [153]. Ecchymosis occurred in the testes of naturally infected Brazilian dwarf brocket deer [154]. Another arbovirus in the genus *Orbivirus* is epizootic hemorrhagic disease virus (EHDV). The virus cycles between *Culicoides* spp. midges and ruminants and often causes fatal hemorrhagic disease in white-tailed deer [155]. EHDV RNA was detected in the testes of naturally infected mule deer, and the virus was implicated as a cause of testicular degeneration [156].

### 1.16. Rhabdoviridae

Seven of the 20 genera in the family *Rhabdoviridae* contain arboviruses, and these are *Curiovirus*, *Ephemerovirus*, *Hapavirus*, *Ledantevirus*, *Sripuvirus*, *Tibrovirus* and *Vesiculovirus*. Bovine ephemeral fever virus (BEFV) is the type species of the genus *Ephemerovirus* and a major pathogen of cattle and water buffalo. The virus was isolated from the semen of one of 12 experimentally infected bulls [157]. Ten heifers artificially inseminated at estrus with semen mixed with virus did not become infected. There is no other data to suggest that arboviruses from this family have the potential to be sexually transmitted between vertebrates.

### 1.17. Togaviridae

The majority of viruses in the genus *Alphavirus* (family *Togaviridae*) are mosquito-borne [158]. The genus includes chikungunya virus (CHIKV), a cause of severe, debilitating and often chronic arthralgia in humans [159]. CHIKV RNA was detected in the semen of a patient with a concurrent DENV infection up to 30 days after symptom onset [72]. Virus isolation was not performed. The patient experienced a burning sensation in the urethra and genital region three days before he developed symptoms characteristic of chikungunya.

Eastern equine encephalitis virus (EEEV) and Highlands J virus (HJV) are also alphaviruses. Both cycle between mosquitoes and passerines and are associated with disease in poultry [158,160]. EEEV and HJV were shed in the semen of domestic turkeys for up to 4 and 5 days post-infection, respectively [161]. EEEV was recovered from one of 10 hens inseminated with virus-contaminated semen. HJV was recovered from three of 10 hens. Evidence of EEEV infection was detected in various tissues of mice and guinea pigs challenged with aerosolized virus, but the testes, ovaries and uterus were negative [162].

Another mosquito-transmitted alphavirus is Venezuelan equine encephalitis virus (VEEV). A live-attenuated vaccine strain of VEEV replicated in the testes of golden hamsters after intratesticular, but not subcutaneous, inoculation [163]. One of 18 females that naturally mated with males inoculated by the intratesticular route seroconverted. Another female died, and virus was isolated from her brain and from one of her progeny.

## 2. Concluding Remarks

Evidence is accumulating that some arboviruses can replicate in the vertebrate reproductive tract and persist in sexual fluids, revealing the important need to investigate further the potential sexual transmission of arboviruses. It is well documented that ZIKV can be transmitted between humans through sexual contact and CCHFV and DENV are potentially transmitted, albeit probably occasionally, by this route [4,5,6,19,20,21,48,49]. Other arboviruses have been detected in human semen by RT-PCR, and these include RVFV, SFTSV and YFV [30,32,55]. Arboviruses detected in tissues and sexual secretions of the human reproductive tract, whether by virus isolation or the identification of viral nucleic acid or protein, include CCHFV, HRTV, WNV and ZIKV [23,28,54,82]. The detection of arboviruses in vertebrate saliva is not discussed because this review is restricted to direct acts of sexual contact.

Evidence also exists that select arboviruses have the potential to be sexually transmitted between vertebrate animals. TBEV and ZIKV have been transmitted between laboratory mice by natural mating and SPONV RNA has been detected in the semen of infected mice [90,93,96,134]. Arboviruses detected in the reproductive tract and/or in sexual fluids of NHPs include CCHFV and ZIKV [24,115,116,117,118,119,120,121,122,123]. Several arboviruses have also been detected in the reproductive tract or sexual fluids of livestock, including AKAV, BEFV, BTV, EHDV, JEV and SBV [37,42,132,143,156,157]. Infections occurred in recipient females artificially inseminated with BTV- and JEV-contaminated semen [132,144,150]. Various arboviruses have also been detected in the reproductive tract or sexual fluids of birds, including EEEV, HJV, Tembusu virus and WNV [130,137,161].

The identification of sexually acquired arbovirus infections is often confounded in geographical regions where vector-borne transmission is known to occur and so care must be taken not to over-interpret data. Sexual transmission was considered the most likely route of infection for a woman with severe WNV disease, but the case occurred in the United States, a WNV endemic region [52]. Although the patient could not recall any recent mosquito bites, she could have unknowingly been bitten by an infected mosquito. The authors’ conclusions should therefore be interpreted with caution. All cases of CCHF suspected to have been sexually acquired occurred in countries where the etiological agent is endemic [19,20,21]. Vertebrate animal studies can be faced with similar problems, as observed in a phenuivirus investigation performed in Australia [43]. Ejaculates from AKAV- and AINOV-infected bulls were tested for virus by sheep inoculation. Several sheep seroconverted but they were housed in a non-insect-proof shed in an area where vector-borne AKAV and AINOV transmission had previously been reported. The authors acknowledged that the sheep could have acquired the infections by insect bite.

Many arboviruses have been detected, either directly or indirectly, in the vertebrate reproductive tract and in sexual fluids. Most experiments were performed using indirect virus detection assays, such as RT-PCR, but this alone is not sufficient evidence that a virus can be sexually transmitted. Virus isolation is more informative but also has its limitations because sexual transmission depends on various factors, including viral dose, the amount of infectious virus in the dose and route of inoculation. Reports of virus RNA in the reproductive tract and sexual fluids of severely ill patients should also be interpreted with care. It may not be all that surprising that viral RNA can enter normally protected sites/fluids like sperm in the testes and seminal fluid in the prostate and seminal vesicles, when tissue integrity is failing. In this situation, virus entry into these sites may not be directly suggestive of a new route of transmission but rather just a symptom of serious disease with the beginnings of organ system failure. Many non-arthropod-borne viruses have been detected in the vertebrate reproductive tract and in sexual secretions, but have never been associated with sexually acquired infections [164].

Various considerations must be taken into account to conclude that an arbovirus can be sexually transmitted and, if it can be, whether it is a rare event or epidemiologically relevant. The importance of developing animal models to study sexual transmission cannot be overstated. Animal models can be used to determine whether viable virus occurs and persists in the male and female reproductive tracts and in their sexual fluids, as done with ZIKV. If virus is detected, it is important to determine whether the amount of virus is enough to infect another host. Different routes of inoculation should be evaluated, including the vaginal and anorectal routes. Natural mating experiments designed to determine whether infected animals can transmit the virus to uninfected animals are especially informative. This work should be performed using infected males and uninfected females, along with reciprocal experiments. One advantage of studying the transmission dynamics of zoonotic diseases and diseases of veterinary importance is that natural animal hosts can be used in the experiments, although it is important that the work is performed under strict vector-protected conditions.

With the exception of ZIKV, sexual transmission appears to have negligible impact on the epidemiology of arboviruses. However, it is possible that other arboviruses could acquire the ability to be efficiently sexually transmitted (i.e., through mutation or reassortment) or that there are undiscovered or poorly studied arboviruses that already possess this trait. If sexual transmission is shown to occur with an arbovirus, it is important to assess its epidemiological relevance and its contribution to transmission in both endemic and non-endemic regions. Sexual and arthropod-borne transmission may act synergistically in vector endemic regions to enhance transmission and maintain endemicity while sexual transmission may not be enough to sustain virus circulation in regions where arthropod vectors are not present. It is also important to determine whether the pathology and symptomatology of sexually acquired infections differ from mosquito-acquired infections. This information will assist in the diagnosis and clinical management of sexually acquired arbovirus disease. If an arbovirus is shown to be sexually transmitted between humans and if this mode of transmission is not a rare event, health authorities should recommend protected sex in populations living in or traveling to endemic countries, especially if there is an ongoing outbreak or epidemic. If efficient arbovirus transmission is shown to occur between livestock or poultry (i.e., through natural mating or artificial insemination), resulting in substantial economic losses, producers need to modify their farming and disease control practices accordingly.

To conclude, many sexually acquired ZIKV infections have occurred in the last decade, and evidence suggests that another major arbovirus pathogen, DENV, could be sexually transmitted, while other arboviruses possess the ability to infect the vertebrate reproductive tract. It is therefore important that additional work be performed to investigate the importance of sexual transmission in the epidemiology, spread and control of arboviruses.

## Figures and Tables

**Table 1:** Arboviruses with the potential to be sexually transmitted between humans as determined by apparent sexually acquired cases or the presence of the virus in the reproductive tract or sexual secretions.

Virus	Number of Reported Cases Suspected to have Occurred by Sexual Transmission	Evidence of the Virus in the Human Reproductive Tract or Sexual Secretions			References
		Virus Isolation	Antigen Detection	Nucleic Acid Detection	
*Bunyavirales*					
Crimean-Congo hemorrhagic fever virus	5 ^a^	NT	NT	+	[19,20,21,22,23]
Heartland virus	0	NT	+	NT	[28]
Rift Valley fever virus	0	−	NT	+	[32]
Severe fever with thrombocytopenia syndrome virus	0	NT	NT	+	[30]
*Flaviviridae*					
Dengue virus	2	NT	NT	+	[48,49,50,51]
West Nile virus	1 ^a^	−	+	+	[52,53,54]
Yellow fever virus	0	NT	NT	+	[55]
Zika virus	Many	+	+	+	[4,5,7,8,9,10,11,12,13,14,47,56,57,58,59,60,61,62,63,64,65,66,67,68,69,70,71]
*Togaviridae*					
Chikungunya virus	0	NT	NT	+	[72]

NT, not tested; +, positive; −, negative. ^a^ Occurred in an endemic region, so the virus could have been acquired by vector-borne transmission.

**Table 2:** Arboviruses with the potential to be sexually transmitted between vertebrate animals as determined by laboratory mating experiments, artificial insemination or the presence of the virus in the reproductive tract or sexual secretions.

Virus	Sexual Transmission between Laboratory Animals	Transmission by Artificial Insemination	Evidence of the Virus in the Reproductive Tract or Sexual Secretions of Vertebrate Animals			References
			Virus Isolation	Antigen Detection	Nucleic Acid Detection	
*Asfarviridae*						
African swine fever virus	NT	+ ^a^	+ ^a^	NT	NT	[16]
*Bunyavirales*						
Aino virus	NT	NT	NT ^b^	NT	NT	[43]
Akabane virus	NT	NT	+	NT	NT	[42]
Crimean-Congo hemorrhagic fever virus	NT	NT	−	+	+	[24]
Schmallenberg virus	NT	NT	+	NT	+	[35,36,37,38,39,40]
*Flaviviridae*						
Japanese encephalitis virus	NT	+	+	+	NT	[132,133]
Spondweni virus	NT	NT	+	NT	+	[87]
Tembusu virus	NT	NT	+	+	+	[135,136,137,138]
Tick-borne encephalitis virus	+	NT	NT	NT	NT	[134]
West Nile virus	NT	NT	NT	+	NT	[130]
Zika virus	+	NT	+	+	+	[90,91,92,93,94,95,96,97,98,99,100,101,102,103,104,105,106,107,108,109,110,111,112,113,114,115,116,117,118,119,120,121,122,123,124,125,126,127]
*Reoviridae*						
Bluetongue virus	−	+	+	+	+	[143,144,145,146,147,148,149,150,151,152,153]
*Rhabdoviridae*						
Bovine ephemeral fever virus	NT	NT	NT	NT	+	[156]
*Togaviridae*						
Eastern equine encephalitis virus	NT	+	+	− ^d^	NT	[161]
Highlands J virus	NT	+	+	NT	NT	[161]
Venezuelan equine encephalitis virus	+^c^	NT	+	+	NT	[163]

NT, not tested; +, positive; −, negative. ^a^ Non-peer-reviewed data. ^b^ Seroconversions occurred in sheep inoculated with ejaculates from viremic bulls. These data could suggest that the ejaculates contained infectious virus, although virus isolation per se was not performed, but the study was performed outdoors in a virus endemic region, so the sheep could have acquired the infections by insect bite. ^c^ The experiments were performed with a live-attenuated vaccine strain of the virus. ^d^ Virus antigen was not detected in the reproductive tract of mice or guinea pigs challenged with aerosolized virus. In another study, virus was isolated from the semen of infected turkeys, and therefore, virus antigen must have also been present. However, antigen detection assays were not performed.
